# Cerium Oxide Nanoparticles with Entrapped Gadolinium for High *T*_1_ Relaxivity and ROS-Scavenging Purposes

**DOI:** 10.1021/acsomega.2c03055

**Published:** 2022-06-07

**Authors:** Peter Eriksson, Anh H.T. Truong, Caroline Brommesson, Anna du Rietz, Ganesh R. Kokil, Robert D. Boyd, Zhangjun Hu, Tram T. Dang, Per O. A. Persson, Kajsa Uvdal

**Affiliations:** † Division of Molecular Surface Physics and Nanoscience, Department of Physics, Chemistry and Biology (IFM), Linköping University, SE-581 83 Linköping, Sweden; ‡ Laboratory of Therapeutic Cellular and Drug Delivery Systems, School of Chemical and Biomedical Engineering (SCBE), Nanyang Technological University, Singapore 637459 Singapore; § School of Pharmacy, Pharmacy Australia Centre of Excellence, The University of Queensland, Brisbane, QLD 4072, Australia; ∥ Division of Plasma Coatings Physics Department of Physics, Chemistry and Biology (IFM), Linköping University, SE-581 83 Linköping, Sweden; ⊥ Division of Thin Film Physics, Department of Physics, Chemistry and Biology (IFM), Linköping University, SE-581 83 Linköping, Sweden

## Abstract

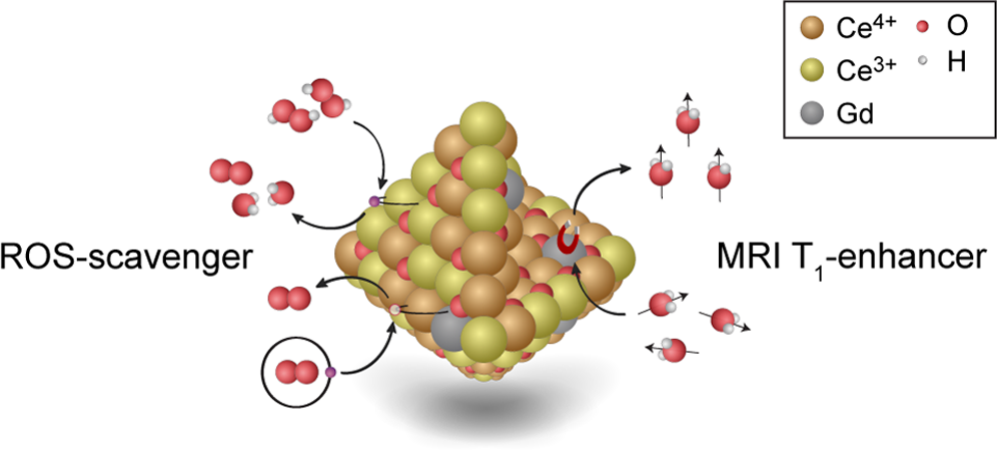

Gadolinium chelates are employed worldwide today as clinical contrast agents for magnetic resonance imaging. Until now, the commonly used linear contrast agents based on the rare-earth element gadolinium have been considered safe and well-tolerated. Recently, concerns regarding this type of contrast agent have been reported, which is why there is an urgent need to develop the next generation of stable contrast agents with enhanced spin–lattice relaxation, as measured by improved *T*_1_ relaxivity at lower doses. Here, we show that by the integration of gadolinium ions in cerium oxide nanoparticles, a stable crystalline 5 nm sized nanoparticulate system with a homogeneous gadolinium ion distribution is obtained. These cerium oxide nanoparticles with entrapped gadolinium deliver strong *T*_1_ relaxivity per gadolinium ion (*T*_1_ relaxivity, *r*_1_ = 12.0 mM^–1^ s^–1^) with the potential to act as scavengers of reactive oxygen species (ROS). The presence of Ce^3+^ sites and oxygen vacancies at the surface plays a critical role in providing the antioxidant properties. The characterization of radial distribution of Ce^3+^ and Ce^4+^ oxidation states indicated a higher concentration of Ce^3+^ at the nanoparticle surfaces. Additionally, we investigated the ROS-scavenging capabilities of pure gadolinium-containing cerium oxide nanoparticles by bioluminescent imaging in vivo, where inhibitory effects on ROS activity are shown.

## Introduction

Linear and macrocyclic gadolinium-based contrast agents (GBCAs) are routinely used as magnetic resonance imaging (MRI) contrast agents in clinics worldwide. These complexes have until recently been considered safe. However, toxicity concerns associated with the use of the commonly used linear GBCAs have been raised.^1,2^ Therefore, there is an urgent need for a new generation of stable, bio-friendly contrast agents with improved contrast and high signal-to-noise properties at low gadolinium (Gd) concentrations. One strategy to reduce the required dose of Gd is to optimize the contrast enhancement per Gd ion through the construction of nanoparticulate systems.^3,4^ Recently, it was shown that when Gd ions are incorporated into nanocrystals, it may improve the rotational correlation time and hydration number. This will enhance the contrast per Gd ion.^5,6^ Furthermore, by guiding the gadolinium-based contrast agents to specific sites by utilizing targeting strategies, the local contrast will be strongly enhanced.^7^ An accumulation of nanoparticles to a specific target results in a high cumulative MR signal from this local region.^8^

These findings pave the way for a new generation of medical treatments, implementing novel concepts including personalized medicine and treatments combined with designs for dedicated targeting and drug release, compared to conventional medicine with standard medical treatments. Nanotechnology continuously contributes to this generational shift, delivering a new medical toolbox for present and future imaging/diagnosis, drug delivery, and treatments. This enables the design of multimodal agents with tailor-made properties for both improved imaging and for advanced treatment combined into a single agent, a so-called theragnostic agent. A novel nanomaterial design plays a key role within this paradigm shift by helping to develop theragnostic treatments.

New promising theragnostic nanoparticles have recently been designed, enabling the diagnosis and treatment of diseases with heterogeneous expressions such as cancer.^9−11^ Other examples include quantum dot–aptamer–doxorubicin for parallel cancer imaging and therapy,^12^ gold-nanoparticle-generated transient photothermal vapor nanobubbles,^13^ and MRI-active iron-based metal–organic frameworks (MOFs) as nanocarriers for antitumoral and retroviral drugs.^14^

Recently, we reported on cerium oxide nanoparticles (CeNPs) with Gd integration exhibiting a strong MR response with the indication of redox properties.^15^ CeNPs are known as strong and recyclable reactive oxygen species (ROS) scavengers by shuttling between Ce^3+^ and Ce^4+^ oxidation states.^16−18^ Their antioxidant properties are dependent on their physical parameters, such as size, agglomeration status in liquid, and surface charge,^19,20^ and on the presence of Ce^3+^ sites at the surface.^21^ The exact mechanism remains to be determined with accurate material characterization being of utmost importance for increasing the understanding of the antioxidant effects in biological systems.

In this study, we report on the synthesis of advanced Gd-CeNPs as well as their inhibitory effect on ROS activity and its correlation to Ce surface states (oxidation number). We have prepared 5 nm sized Gd-CeNPs of high crystal quality, showing strong 1/*T*_1_ relaxivity. The concentrations of Ce^3+^ and Ce^4+^ in the core vs the shell were obtained by scanning transmission electron microscopy with electron energy loss spectroscopy (STEM-EELS) analysis, specifically their relative contributions to the Ce M_5,4_ edge. The ROS-scavenging properties were investigated in vivo using a luminol assay for pure CeO*_x_* NPs and CeO*_x_* NPs with Gd ions incorporated.

## Results and Discussion

**Figure 1:**
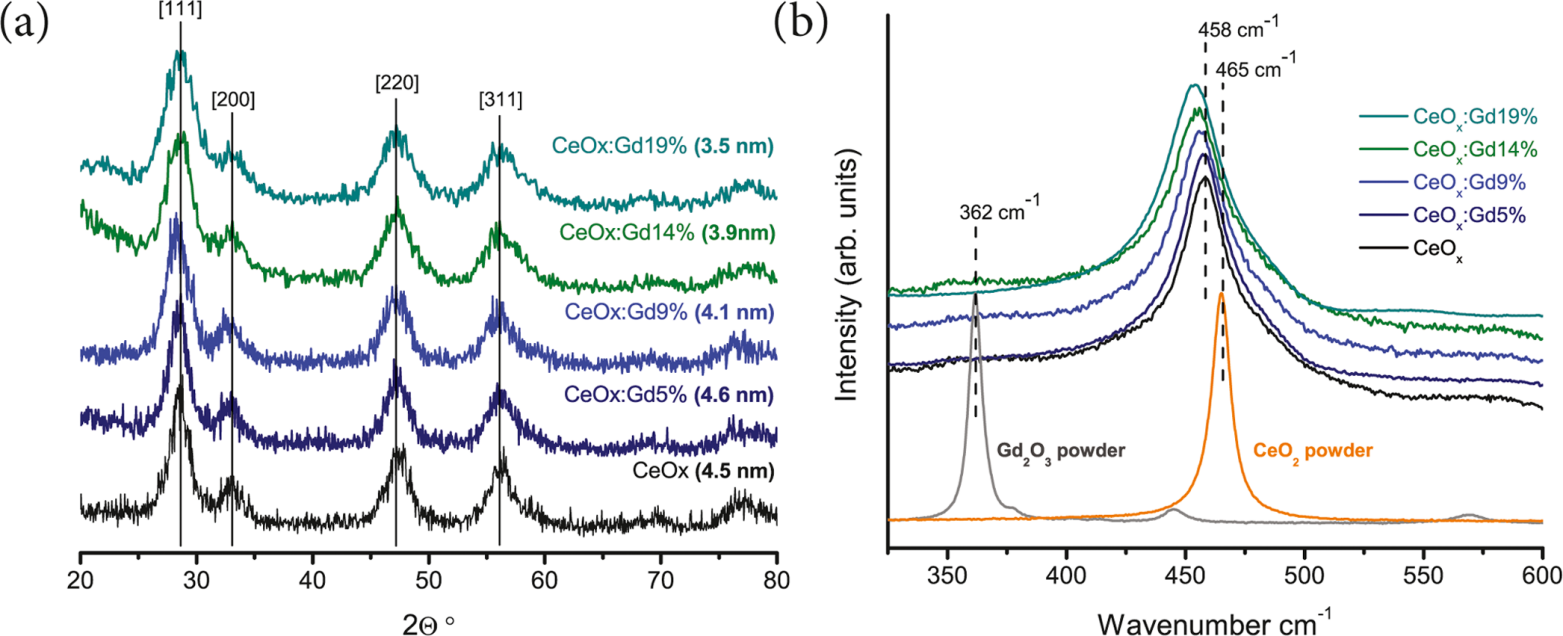
(a) X-ray diffraction patterns of CeO*_x_*, CeO*_x_*:Gd5%, CeO*_x_*:Gd9%, CeO*_x_*:Gd14%, and CeO*_x_*:Gd19%. The calculated grain sizes as determined by the Scherrer equation are given. (b) Raman spectra for CeO_2,_ Gd_2_O_3,_ CeO*_x_*, CeO*_x_*:Gd5%, CeO*_x_*:Gd9%, CeO*_x_*:Gd14%, and CeO*_x_*:Gd19%. The Raman spectra are normalized and vertically shifted using a small offset for each spectrum to facilitate line shape comparison. There is an increasing shift of the main Raman peak to lower wavenumbers related to the integration of Gd.

We have synthesized a set of CeO*_x_* NPs with Gd ions incorporated (CeO*_x_* Gd NPs) using a wet-chemistry-based method. The obtained CeO*_x_*, CeO*_x_*:Gd5%, CeO*_x_*:Gd9%, CeO*_x_*:Gd14%, and CeO*_x_*:Gd19% (the amount of Gd is denoted in %) were synthesized and carefully characterized. The atomic composition of the synthesized Gd-CeNPs was obtained using inductively coupled plasma mass spectrometry (ICP-MS). The physical properties such as crystallinity, size, and size distribution were investigated, and elemental compositions were characterized. The X-ray diffraction (XRD) patterns from the synthesized Gd-CeNPs are presented in Figure 1a, and all display the characteristic peaks of a cubic fluorite structure of cerium oxide, corresponding to the [111], [200], [220], and [311] crystalline planes.^22^ An estimate of the representative size for each set of Gd-CeNPs was calculated from the XRD and TEM results. The XRD peaks broaden and shifted upon Gd incorporation above 5% corresponding to a reduced particle size,^23^ which was estimated through the Scherrer equation. Such XRD peak broadening due to reduced size is consistent with previously published results.^15^ Deshpande et al have shown that decreasing the size of CeNPs correlates to an increasing lattice parameter and a Ce^3+^/Ce^4+^ ratio.^24^ Herein, when Gd ions are incorporated into the NPs, this induces an alteration in the oxidation state of Ce from 4+ to 3+^15^ and consequently a reduction of the lattice parameter.

Dynamic light scattering (DLS) and ζ potential measurements were used to investigate the hydrodynamic diameter and stability of the nanoparticle’s suspensions in an aqueous environment, respectively. CeO*_x_*:Gd0–19% samples were readily dispersible in water, displaying hydrodynamic diameters of less than 7 nm, and ζ potentials obtained were above 30 mV required to ensure a stable dispersion. The detailed results on DLS and ζ potentials are presented in the Supporting information (see Figures S1 and S2).

Next, Raman spectroscopy was used to obtain structural information from the full set of CeO*_x_*:Gd0–19% nanoparticles as compared to powder samples for pure cerium and gadolinium oxide nanoparticles (see Figure 1b). The Raman spectra for the Gd_2_O_3_ powder sample display a main characteristic peak at 362 cm^–1^, which can be attributed to the C-type structure (6-fold coordination, space group *Ia*3, (Th7)), while for CeO_2_ (powder sample), it is found at 465 cm^–1^, which can be attributed to the F-type structure (8-fold coordination, space group *Fm*3*m*, (Oh5)).^25^ Raman spectra for the as-prepared Gd-CeNPs display a single F2g peak, indicating that Gd is well incorporated in the F-type structure of CeNPs for all samples. These results are consistent with previously published studies by Banerji et al and Godinho et al.^25,26^ The F2g peak for the as-prepared Gd-CeNPs was shifted toward lower reciprocal values with increasing Gd content and displayed asymmetrical line shapes and a broader linewidth compared to the reference sample of cerium oxide. The two Raman spectra of CeO*_x_* and CeO*_x_*:Gd5% nanoparticles exhibit a peak at 458 cm^–1^ assigned to F2g, while a red shift to lower reciprocal values is observed for the CeO*_x_*:Gd9–19% sample. These findings are in good agreement with results reported by Spanier et al,^27^ who studied the Raman F2g peak of CeNPs and observed a red shift as well as linewidth broadening and asymmetrical line shape upon size reduction rather than from Gd incorporation. These peak shifts are caused by several contributing factors, including phonon confinement, strain, broad size distributions, defects, and variations in phonon relaxation with particle size.^27^

**Figure 2:**
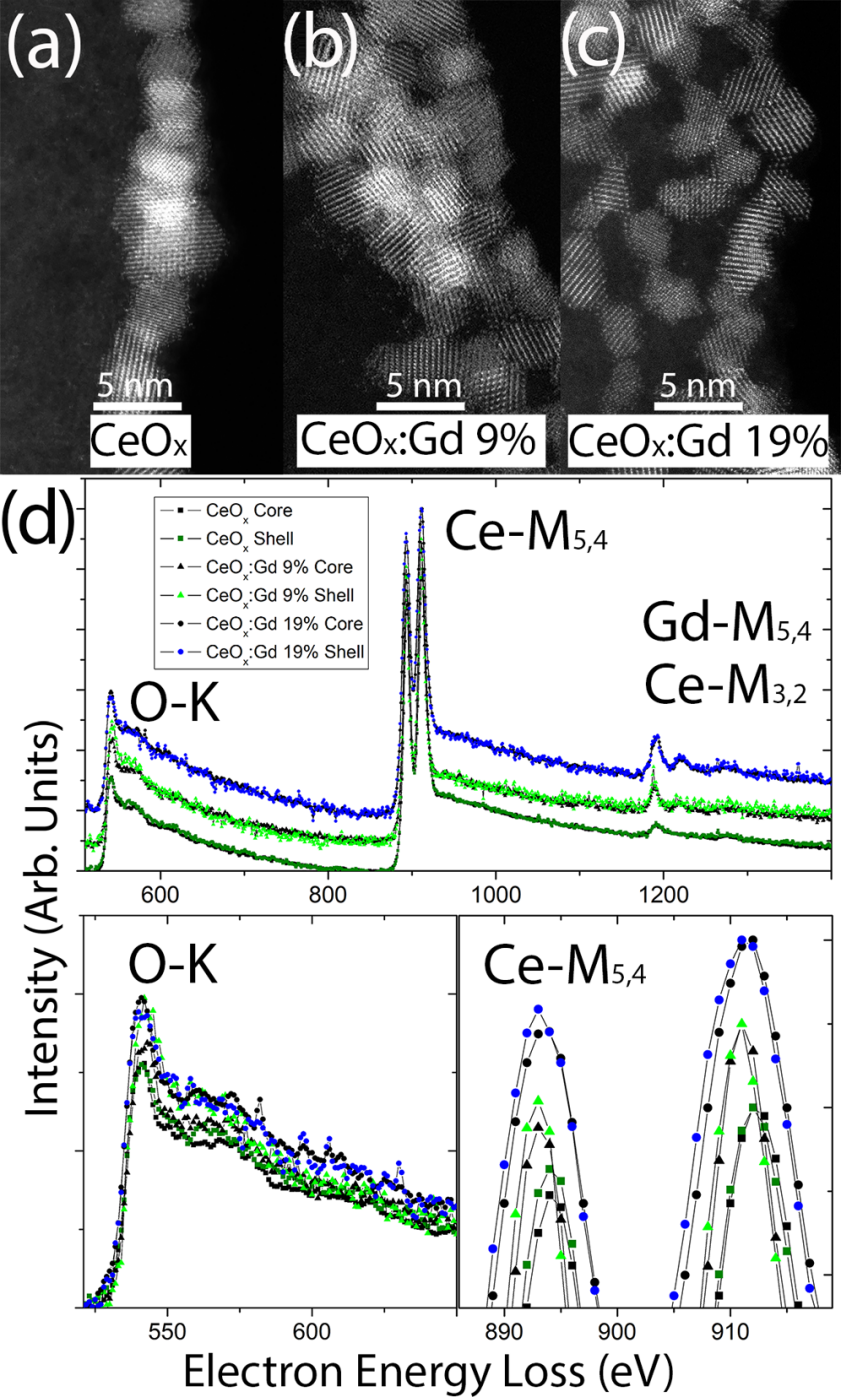
(a–c) High-resolution HAADF-STEM images of the CeO*_x_*, CeO*_x_*:Gd9%, and CeO*_x_*:Gd19% particles, respectively. (d) Vertically shifted core-loss EEL spectra (top) for the same samples, with emphasis on baseline aligned the O-K (bottom left) and vertically shifted Ce-M (bottom right) edges.

STEM-EELS was used to spatially resolve the structural and chemical properties of the nanoparticles. The dispersed particles are shown in Figure 2a–c for CeO*_x_*, CeO*_x_*:Gd9%, and CeO*_x_*:Gd19%, respectively. All mass-sensitive images show nanoparticles with sizes at about 4 nm, exhibiting no apparent compositional modulation within the particles. EELS spectrum imaging (SI) was performed to obtain spatially resolved information on the oxidation state in the core and shell region of the particles.

Figure 2d shows the EEL spectra from CeO*_x_*, CeO*_x_*:Gd9%, and CeO*_x_*:Gd19, where the Gd-containing compositions are vertically shifted from CeO*_x_* at the bottom with CeO*_x_*:Gd19% at the top. For each composition, the core spectrum (black) and shell spectrum (colored) are superimposed. The overview spectra in 2 days contain information from the O-K, Ce-M, and Gd-M edges, and the spectra have been normalized against the maximum intensity of the Ce-M_4_ edge. The increase in Gd content is therefore visible by the increase in edge intensity at ∼1200 eV. Note that the Ce-M_3,2_ and Gd-M_5,4_ edges are superimposed, which is why there appears to be a Gd signal in the CeO*_x_* spectra. The relative increase of Gd is associated with a corresponding decrease in Ce. Since the edge intensities are normalized against the Ce-M_4_ edge, the O-K edge increases with Gd content. This can be observed in the detailed O-K edge, where all spectra are aligned for this purpose. The O content in shell vs core cannot be accurately assessed from the O-K edge due to the signal-to-noise ratio; however, the Ce-M_5,4_ edge shows differences in oxidation state. The peaks of the Ce-M_5,4_ edge have been magnified in Figure 2d (bottom right), where compositions remain vertically separated and intensities remain normalized with respect to Ce-M_4_. To verify the difference in the oxidation state between the shell and core, we compare the peak heights of the Ce-M_5_ spectra from the shell (colored) vs core (black). It is clear that the shell spectra exhibit a slightly stronger intensity, both in terms of peak height and area, compared to the core spectra, which indicate that the Ce atoms of the nanoparticle shells exhibit a lower valence state than the core atoms.^28^ The spectra obtained from the particle cores contain a non-negligible component from the shell, since the transmitted electron beam passes through the particle, thereby interacting with both “top” and “bottom” of the shell as well as with the core. Therefore, the measured core Ce-M_5_ intensity is artificially increased. Despite this, it remains lower than the shell Ce-M_5_ intensity.

**Figure 3:**
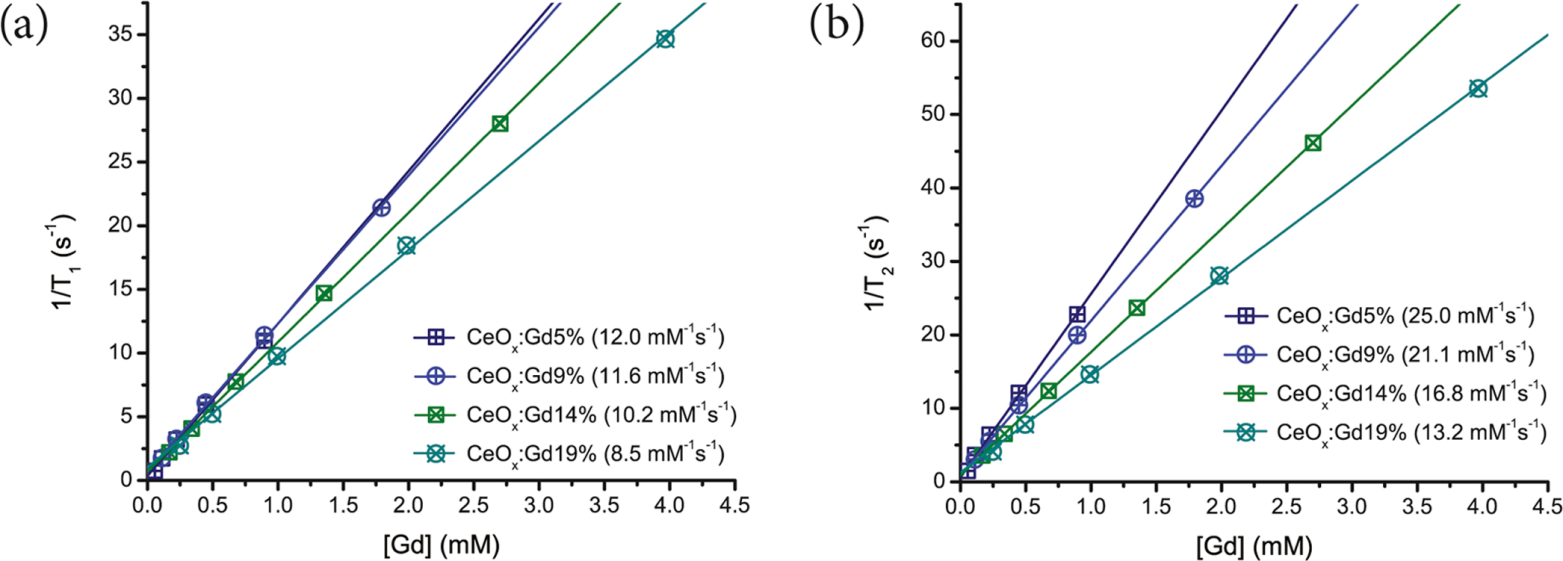
Inverse of relaxation times (a) 1/*T*_1_ and (b) 1/*T*_2_ are shown as a function of the concentration of gadolinium for the prepared Gd-CeNPs. The slope of the fitted linear equations denoted as the *r*_1_ (a) and *r*_2_ (b) relaxivities are given within the brackets.

To further investigate the potential for contrast enhancement, we measured the *T*_1_ and *T*_2_ relaxivities (*r*_1_ and *r*_2_), which relates to the intrinsic ability of an MRI contrast agent to deliver positive and negative contrast, respectively. The relaxivity values *r*_1_ and *r*_2_ for CeO*_x_*:Gd5–19% are presented in Figure 3. Both *r*_1_ and *r*_2_ decrease with higher Gd integration. However, *r*_2_ decreases more rapidly meaning that the *r*_2_/*r*_1_ ratio is decreasing. A *T*_1_-weighted contrast agent for MRI has a typical *r*_2_/*r*_1_ ratio equal to or below 2;^29^ therefore all of the prepared Gd-CeNPs are considered as positive contrast agents. The *r*_1_ value is the dominant factor for *T*_1_-weighted contrast enhancement^30^ and is the most interesting parameter for positive MRI contrast agents.

CeO*_x_*:Gd5–9% has the highest *r*_1_ relaxivity between 12.0 and 11.6 mM^–1^ s^–1^. Therefore, the Gd-CeNPs with Gd content <10% are considered to be the most promising for MRI applications.^15^ The *r*_1_ is clearly decreasing for Gd-CeNPs with a Gd ratio above 10%, and in the present work, we prove that the contrast efficiency per Gd ion decreases for CeO*_x_*:Gd ≥ 14% nanoparticles. These *r*_1_ values are higher than those reported for Gd_2_O_3_ nanoparticles of various sizes^4,5^ and about three times higher than the commercially available positive MRI contrast agents.^31^

**Figure 4:**
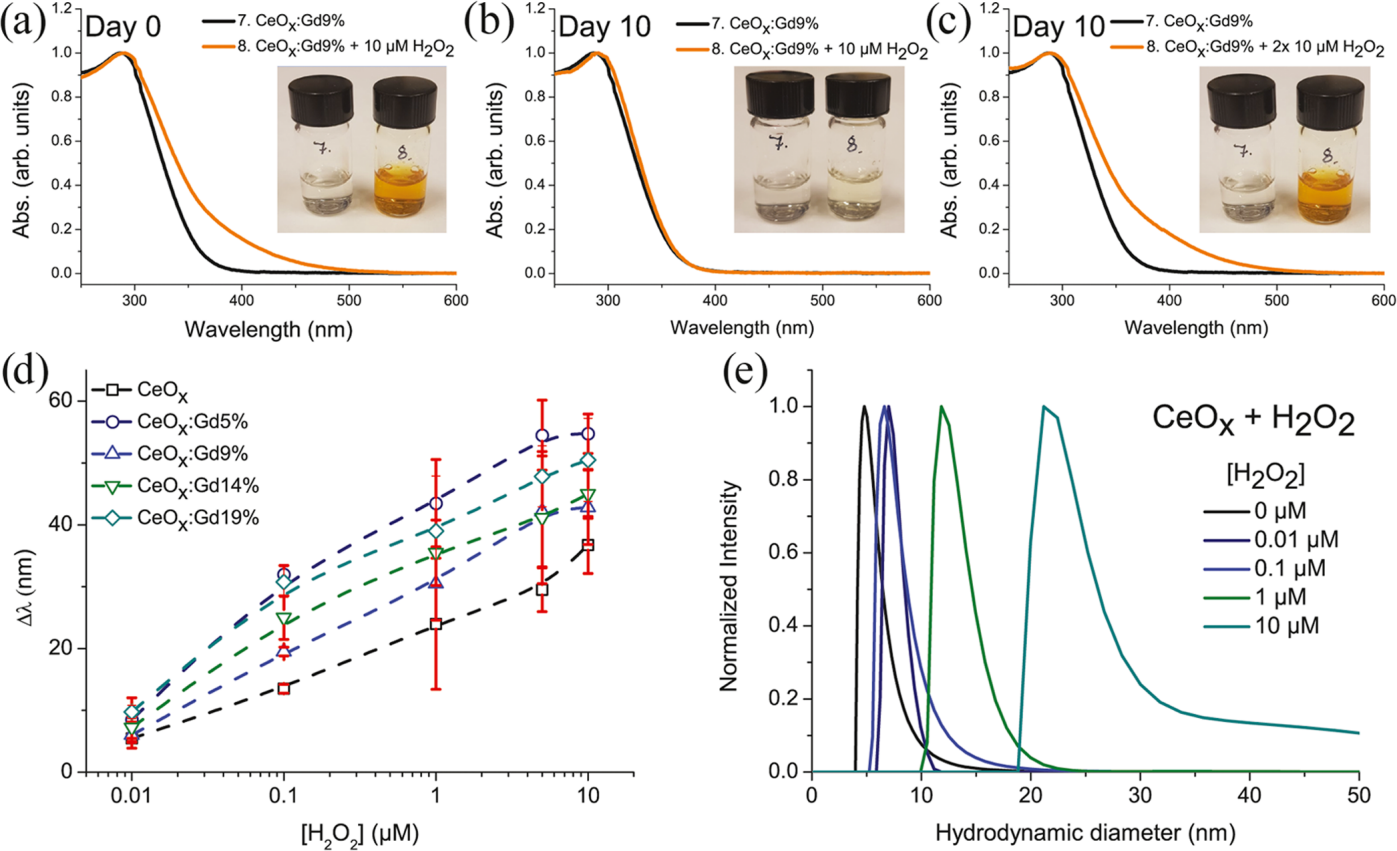
Absorbance spectra and inserted images of CeO*_x_*:Gd9% untreated and treated with 10 μM H_2_O_2_ on (a) day 0, (b) day 10, and (c) on day 10, another 10 μM H_2_O_2_ was added to the sample. (d) Plotted red shifts CeO*_x_*:Gd0–19% ([Ce] = 10 μg mL^–1^) treated with increasing concentration of H_2_O_2_. (e) Hydrodynamic diameter of CeO*_x_* treated with H_2_O_2_ (number-weighted distributions). The photos in panels (a)–(c) courtesy of the first author, P. Eriksson. Copyright 2020.

The Raman and STEM-EELS results are consistent with Gd homogeneously distributed within the as-prepared Gd-CeNPs. The remarkable high relaxivities could not be explained by favorable Gd localization on the surface. It should be noted that cerium oxide has been reported to have a unique contribution to the association and dissociation of water in inverse catalyst systems.^32^ The exchange of water in the proximity of Gd will affect the relaxivity of a contrast agent. The catalytic redox properties, an indication of ROS-scavenging effects, of Gd-CeNPs can be demonstrated by spectral and visual color changes upon mixing with H_2_O_2_ (see Figure 4a–c). The Gd-CeNPs change quickly from transparent to intense yellow color when mixed with H_2_O_2_, leading to a red shift in the UV–vis absorbance spectrum. After 10 days of incubation, the suspension recovers to its original transparency. Upon new hydrogen peroxide incubation, there is a red shift of the Gd-CeNP solution again showing an intense yellow color. The color change is attributed to the oxidation of Ce^3+^ surface ions on the surface to Ce^4+^ by H_2_O_2_^18^ or/and the formation of coordinated peroxide species on Gd-CeNP surfaces.^33,34^ We characterized the red-shift absorbance by plotting the wavelength difference Δλ at an optical density of 0.05 (see, for example, Figure S3 Supporting information) for several concentrations of H_2_O_2_ (see Figure 4d). The shifted value is dependent on the specific H_2_O_2_ concentration.^35^ For all samples, the wavelengths are continuously shifted for increasing H_2_O_2_ concentration up to 5–10 μM. The H_2_O_2_-treated nanoparticles studied by DLS displayed a stable hydrodynamic diameter within the range 5–30 nm. Indications of aggregations were observed for particles treated with H_2_O_2_ at concentrations above 50 μM (Figure 4e). Future surface functionalization may prevent such aggregation, in line with our previous polymer-capsulated gadolinium-based nanoparticles, see Ahrén et al^3^ and Hu et al.^36^ Lee et al demonstrated the correlation of red shift and antioxidant capacity;^18^ therefore, we consider CeO*_x_*:Gd5–19% to have slightly better antioxidant capacity than pure CeO*_x_*.

**Figure 5:**
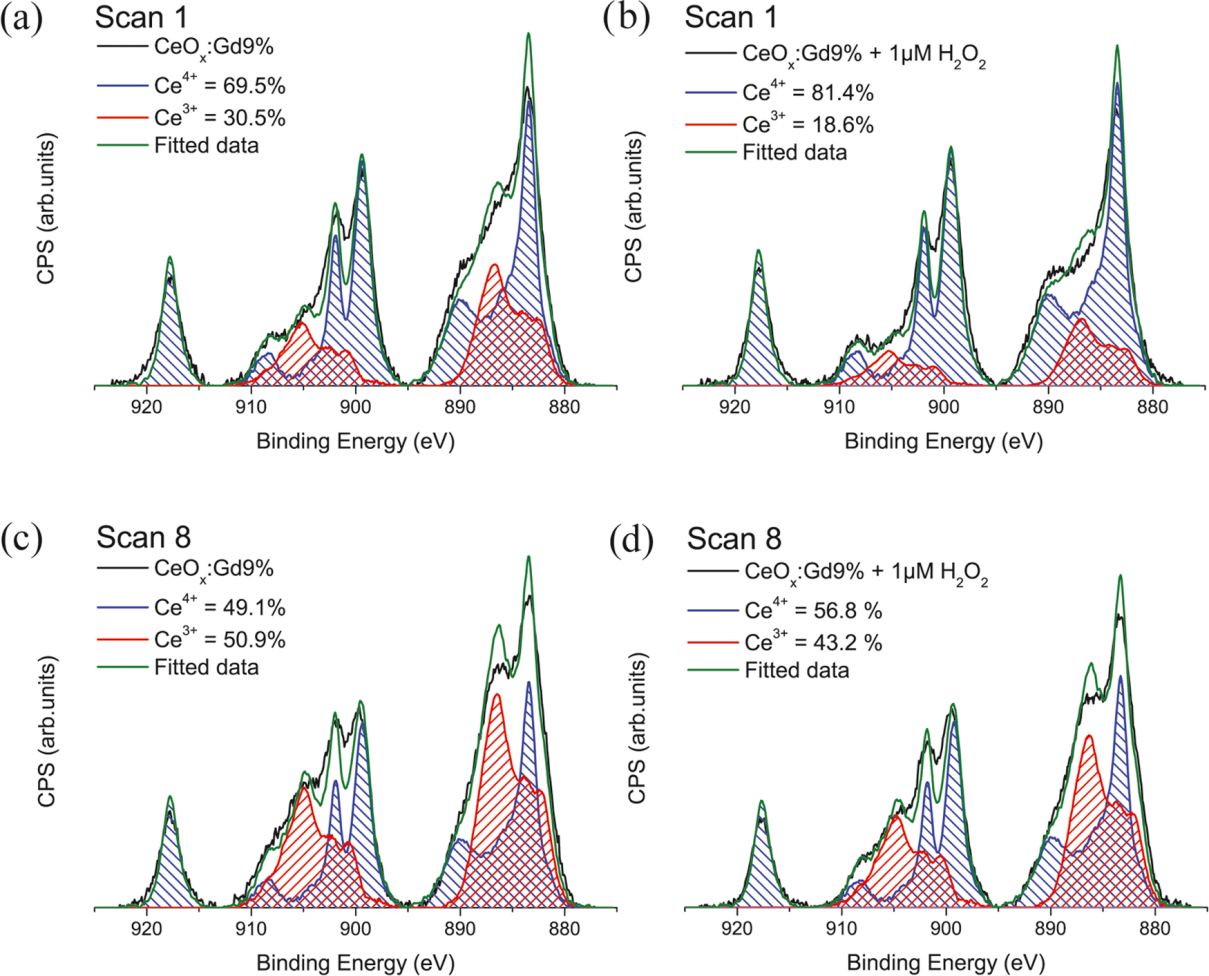
XPS spectra of (a) CeO*_x_*:Gd9% scan 1, (b) CeO*_x_*:Gd9% treated with 1 μM H_2_O_2_ scan 1, (c) CeO*_x_*:Gd9% scan 8, and (d) CeO*_x_*:Gd9% treated with 1 μM H_2_O_2_ scan 1.

Analysis of Ce 3d XPS spectra is a common approach to quantify the oxidation states of CeNPs^37−39^ despite the issue of CeNPs changing in oxidation states upon X-ray radiation.^28^ By following the procedure of Baltrusaitis et al,^40^ we have analyzed the Ce^3+^ proportions for our CeO*_x_*:Gd9% treated and untreated with 1 μM H_2_O_2_. A Ce^3+^ proportion shift from 30.5 to 18.6% (Figure 5a,b) was observed, indicating an active antioxidant material.^18^ Repeating the scan on the same CeO*_x_*:Gd9% sample demonstrates how quickly the Ce^3+^/Ce^4+^ proportion increases upon X-ray exposure (Figure 5c,d). Care must be taken with any X-ray radiation of CeNPs prior to XPS analysis.

In this study, where CeO*_x_* is chosen as a carrier for Gd, Ce delivers antioxidant behavior and Gd delivers MR contrast. When designing nanoparticles with strong MR contrast properties and intrinsic antioxidant properties combined, a tradeoff must be made between the two. Earlier results indicate that a ratio equal to or less than 9% Gd (<10%) is the most promising for future applications (antioxidant MR probes).

**Figure 6:**
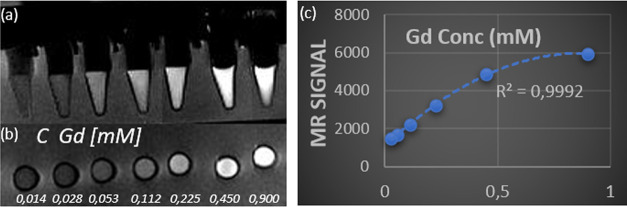
*T*_1_-weighted MR images of CeO*_x_*:Gd9% nanoparticles as a function of Gd concentration: (a) schematic image side view of measured tubes, (b) top view, and (c) measured MR signal (*T*_1_-weighted) from the cylindrical volume of 3 mm height and 5 mm diameter.

*T*_1_-weighted MR imaging was performed to confirm the *T*_1_ ability of CeO*_x_*:Gd5% nanoparticles. MR imaging was obtained with a 4T MRI scanner using a three-level phantom model of our own design (see Figure S4). All samples were measured in a Milli-Q water-filled phantom kept at human body temperature. Dilution series of CeO*_x_*:Gd5% nanoparticles were prepared and the *T*_1_-weighted images were recorded. MR images, side view and top view, of thin slices are shown in Figure 6a,b. The total signal from the cylindrical volume of 3 mm height, and 6 mm diameter for each sample of the dilution series was recorded, and the total *T*_1_-weighted MR signal for the dilution series was plotted as a function of Gd concentration (see Figure 6c).

**Figure 7:**
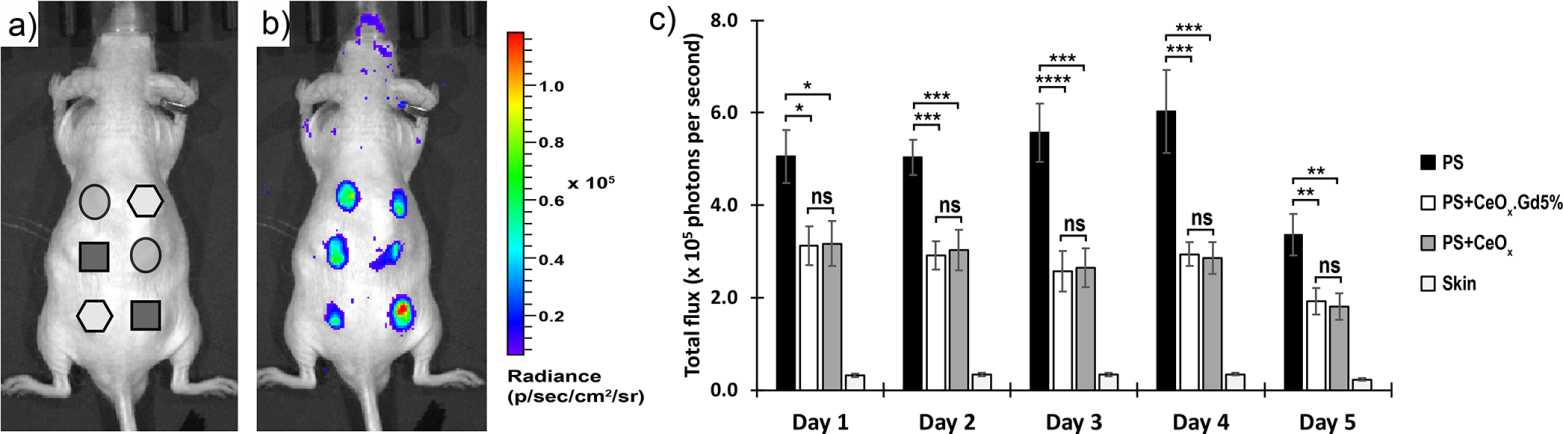
Antioxidant behavior of CeO*_x_* and CeO*_x_*:Gd5% nanoparticles in immunocompetent mice. (a) Schematic of subcutaneous injection on the dorsal side of a mouse showing positions of different particle formulations including a mixture of CeO*_x_* with PS (hexagonal), a mixture of CeO*_x_*:Gd5% with PS (circle), and PS microparticles only (square) as a control. (b) Bioluminescent image of the ROS signal from a representative mouse on day 3, following subcutaneous injection of particles. (c) Quantification of bioluminescent signals from injection sites over a 5 day period. Error bars are standard of the mean (*n* = 12 injections on 6 mice with 2 replicates per mouse). *, ^**^, ^***^, ^****^ denote *p* ≤ 0.05, 0.01, 0.001, and 0.0001, respectively. “ns” denotes nonsignificance, which has *p* > 0.05.

We performed in vivo studies to verify the antioxidant properties of Gd-CeNPs. CeO*_x_* and CeO*_x_*:Gd5% samples were selected based on our previously performed in vitro antioxidant assay.^15^ A mouse model was used^41−43^ with three particle suspensions, a mixture of polystyrene (PS) microparticles and CeO*_x_*, a mixture of PS microparticles and CeO*_x_*:Gd5%, and a control of PS microparticles only, subcutaneously injected separately into each mouse, following the schematic pattern given in Figure 7a. We evaluated ROS levels by quantification of bioluminescent intensity, which was generated due to the ROS-mediated oxidation of luminol.^43,44^Figure 7b shows the bioluminescent images of luminol (i.e., ROS signal) from a representative mouse on day 3, following subcutaneous injection of the particle mixture. Quantification of the bioluminescent signals (Figure 7c) indicated that the ROS activity at the injection sites with CeO*_x_* or CeO*_x_*:Gd5% was significantly lower compared to the site with control PS microparticles only. Interestingly, the inhibitory effect on ROS activity due to the presence of CeO*_x_* or CeO*_x_*:Gd5% persisted over the entire 5 day period of study even though the nanoparticles were only injected once, together with the PS microparticles, on day 0. The background signal from the skin region adjacent to the sites with injected PS microparticles was negligible compared to that of the sites with PS microparticles only. There was no significant difference in the ROS signal between CeO*_x_* and CeO*_x_*:Gd5%, but the present results confirmed the antioxidant properties of CeNPs using a relevant in vivo model. In addition, the effect persisted even 5 days postinjection, indicating a potential for modulation of ROS after the initial acute inflammatory phase.

## Conclusions

In this study, we have synthesized and characterized 5 nm sized pure cerium oxide nanoparticles (CeNPs) with increasing content of gadolinium (Gd) up to 19%. All prepared nanocrystals exhibit a cubic fluorite structure and Gd is homogeneously distributed throughout Gd-CeNPs. The radial distribution of Ce^3+^ and Ce^4+^ oxidation states shows a higher concentration of Ce^3+^ at the particle surface. It is suggested that oxygen vacancies facilitate the transformation/cycling between Ce^4+^ and Ce^3+^, which may be the fundamental principle behind many of the useful properties of CeNPs. A strong 1/*T*_1_ relaxivity was obtained and the presence of crystalline CeNPs has an inhibitory effect on ROS activity, i.e., can scavenge ROS efficiently to reduce oxidative stress in vivo*.* Our results demonstrate a novel strategy for the development of crystalline nanoprobes for enhanced 1/*T*_1_ relaxivity with increased local contrast and scavenger capability of reactive oxygen species (ROS), which has important implications for the next generation of MR contrast agents. Detailed future experimental and theoretical studies may contribute to their formulation by providing information on the role of oxygen vacancies in stabilizing the surface states.

## Materials and Methods

### Synthesis and Purification of Nanoparticles

Gd-CeNPs were prepared with 0–19 mol % Gd content, utilizing a simple wet-chemical-based procedure at room temperature. All solutions used in the synthesis were pumped and purged with nitrogen gas. First, 0.5 mmol of cerium(III)- and gadolinium(III)-acetate were dissolved in 10.96 mL of a 50/50 Milli-Q water and diethylene glycol (DEG) solution. DEG (50/50, 1.04 mL) and 30% ammonium hydroxide were added dropwise to the solution under constant stirring and N_2_ gas flow. The solutions were kept under constant stirring and N_2_ gas flow for 2 h until the syntheses were stopped. Thereafter, the prepared Gd-CeNPs were dialyzed (Slide-A Lyzer G2 Dialysis Cassettes, 20 K MWCO, 15 mL) against Milli-Q water at a minimum ratio of 1:1000 for 24 h with two water exchanges. After dialysis, the nanoparticle solutions were filtered using an Acrodisc 25 mm syringe filter with w/0.1 μm supor membrane.

### Inductively Coupled Plasma Mass Spectrometry (ICP-MS)

The ICP-MS measurements were performed by ALS Scandinavia AB.

### X-ray Diffraction (XRD)

Powder samples of Gd-CeNPs for XRD were obtained by lyophilization and were examined with a Phillips PW 1820 powder diffractometer using Cu Kα radiation (λ = 1.5418 Å, 40 kV, 40 mA).

### Relaxivity

The relaxivity study was carried out using a Bruker minispec mq60 NMR analyzer (40 °C, 1.41 T). Samples were diluted using Milli-Q water and temperature-stabilized for at least 4 min prior to measurements.

### Magnetic Resonance Imaging (MRI)

The sequence used is the spin-echo sequence. The TR (repetition time) is 550 ms, and TE (echo time) is 10 ms for the *T*_1_-weighted images. The section thickness is 3 mm and FoV is 200 mm.

Samples were prepared and installed in a prototype sample holder, see Figure S4, that was put into a container filled with water kept at human body temperature. All experiments were performed on a 3T clinical scanner (Ingenia, Philips Healthcare, Best, The Netherlands) with a 28-channel torso coil.

### ζ Potential

ζ Potential measurements were performed on a Malvern Zetasizer Nano ZS90 operated at 25 °C using DTS1070 cuvettes.

### Dynamic Light Scattering (DLS)

Dynamic light scattering measurements were performed on an ALV/DLS/SLS5022F system (ALV-GmbH, Langen, Germany) using a HeNe laser at 632.8 nm, operating at 20 °C and measuring at 90° scattering angle. The samples were thermally stabilized in a thermostat bath at 20 °C for 15 min before measurement. The contin analysis model was utilized to fit the correlation curve since the polydispersity index was 0.3–0.4 for the as-prepared Gd-CeNPs.

### Raman Spectroscopy

Raman samples were prepared by drying Gd-CeNPs onto a gold surface (10 mm × 15 mm) under N_2_ gas flow. The measurements were performed at room temperature in a backscattering geometry using a 660 nm excitation of a solid-state laser. The laser beam was focused onto the sample with a spot diameter of 0.5–1 μm utilizing a 100× (0.9 NA) objective lens. The signal was dispersed using a single-grating monochromator and collected using a Si CCD.

### Scanning Transmission Electron Microscopy-Electron Energy Loss Spectroscopy (STEM-EELS)

STEM-EELS was performed in a Linköping double aberration-corrected, monochromated, high brightness G2 Titan^3^ 60–300, equipped with a quantum ERS GIF. The EEL signal-to-noise ratio was optimized for 60 pA beam current, so as not to affect the structure. The STEM convergence semiangle was 20 mrad with a corresponding collection angle. Spectra were collected simultaneously in low- and core-loss mode and analyzed using plural scattering deconvolution and background subtraction using the built-in routines implemented in a digital micrograph.

### Optical Spectroscopy

The absorbance spectra were recorded 3 h after the reaction (stored dark and at 4 °C) using a Shimadzu UV-2450 spectrophotometer with a spectral resolution of 0.5 nm. The spectra were subtracted with acquired H_2_O_2_ spectra for corresponding concentration. Δλ was measured at an optical density of 0.05.

### X-ray Photoelectron Spectroscopy (XPS)

XPS measurements were carried out using a VG microlab Auger spectrometer with a 310-F analyzer using unmonochromatized Al Kα photons (1486.6 eV). The energy resolution was approximately 1.9 eV for the experimental settings used, as determined from the full width at half-maximum of the peak-fitted Au 4f_7/2_ line. Each Ce 3d scan had a pass energy of 20 eV, step length of 0.1 eV, and dwell time of 500 ms. The nanoparticle samples were deposited on a TL-1 cleaned gold substrate. The acquired Ce 3d spectra were aligned to the Au 4f_7/2_ peak (84.0 eV), and the photo cross section for the gold substrate in the Ce 3d region was subtracted.

XPS measurements of the reference samples Ce(III)acetate and Ce(IV)oxide nanopowder were carried out using an AXIS UltraDLD instrument from Kratos Analytical and analyzed with monochromatic Al Kα (1486.6 eV) radiation. Energy resolution for the experimental settings was determined to be 0.8 eV, utilizing full width at half-maximum of the peak-fitted Au 4f_7/2_ line. The samples were drop-casted on a TL-1 cleaned gold substrate.

The gold substrates were produced by evaporating 2000 Å gold onto a (111) Si surface precoated with a 25 Å thick layer of Ti.

### Animal Model and Ethical Considerations

The animal protocol (A0343) was approved by the local animal ethics committee at the Nanyang Technological University (Committee on Animal Care, Singapore) prior to the initiation of the study. The 36-week-old male SKH-1E hairless immunocompetent mice were bred and housed under standard conditions with a 12 h light/dark cycle at the animal facilities. The SKH-1E mice parents were purchased from Charles River Laboratories.

### Subcutaneous Injection of Microparticles and Nanoparticles

Before subcutaneous injection, PS microparticles of 6 μm in diameter (Spherotech) were washed with 100% ethanol and 70% ethanol, followed by a final wash with water before resuspension in 0.1 M HEPES buffer. The mice were anesthetized by inhalation of 2% isoflurane in oxygen at a flow rate of 2.5 L min^–1^ before subcutaneous material injection. A 100 μL suspension of 5 mg of PS microparticles with 40 μL of CeO*_x_* ([RE] = [Ce] + [Gd] = 20 mM) or 40 μL of CeO*_x_*:Gd5% ([RE] = 20 mM) nanoparticles was injected subcutaneously at each of the six spots on the dorsal side of each immunocompetent mouse on day 0. Each material formulation was injected at two spots on each mouse.

### Noninvasive Bioluminescence Imaging of SKH-1E Mice

Reactive oxygen species (ROS) were detected by daily imaging of the bioluminescent signal, which is generated by ROS-induced oxidation of luminol (5-amino-2,3-dihydro-1,4-phthalazine-dione; Sigma-Aldrich). During the imaging procedure, the mice were anesthetized by 2.5% isoflurane in the presence of oxygen flow. A 100 μL volume of luminol, dissolved in PBS at a concentration of 50 mg mL^–1^, was intraperitoneally injected into each mouse 20 min before imaging. Noninvasive bioluminescence imaging was performed using a IVIS-spectrum CT system (Perkin Elmer) with a 3 min exposure time. Bioluminescent images were analyzed using Living Image 3.1 software. Similar regions of interest (ROIs) were found close to the injection spots. ROI signal intensities were calculated in total flux (photons/second).

### Statistical Analysis

The animal experiment was repeated on six mice. The data were averaged and represented as the mean ± standard error of the mean. ROS level comparison between different material formulations was performed using one-way ANOVA analysis with Tukey’s multiple comparison test. Measurements with *p*-values less than 0.05 were considered significant.

## Supporting Information Available

ζ potential and dynamic light scattering; UV–vis absorbance; three-level phantom model for MRI studies (PDF)

## References

[ref1] Todd D. J.; Kay J. Nephrogenic systemic fibrosis: An epidemic of gadolinium toxicity. Curr. Rheumatol. Rep. 2008, 10, 195–204. 10.1007/s11926-008-0033-6. 18638427

[ref2] Sharma P.; Brown S.; Walter G.; Santra S.; Moudgil B. Nanoparticles for bioimaging. Adv. Colloid Interface Sci. 2006, 123–126, 471–485. 10.1016/j.cis.2006.05.026. 16890182

[ref3] Ahrén M.; Selegård L.; Klasson A.; Söderlind F.; Abrikossova N.; Skoglund C.; Bengtsson T.; Engström M.; Käll P.-O.; Uvdal K. Synthesis and Characterization of PEGylated Gd2O3 Nanoparticles for MRI Contrast Enhancement. Langmuir 2010, 26, 5753–5762. 10.1021/la903566y. 20334417

[ref4] Ahrén M.; Selegård L.; Söderlind F.; Linares M.; Kauczor J.; Norman P.; Käll P.-O.; Uvdal K. A simple polyol-free synthesis route to Gd2O3 nanoparticles for MRI applications: an experimental and theoretical study. J. Nanopart. Res. 2012, 14, 1006 10.1007/s11051-012-1006-2.

[ref5] Park J. Y.; Baek M. J.; Choi E. S.; Woo S.; Kim J. H.; Kim T. J.; Jung J. C.; Chae K. S.; Chang Y.; Lee G. H. Paramagnetic Ultrasmall Gadolinium Oxide Nanoparticles as Advanced T1 MRI Contrast Agent: Account for Large Longitudinal Relaxivity, Optimal Particle Diameter, and In Vivo T1 MR Images. ACS Nano 2009, 3, 3663–3669. 10.1021/nn900761s. 19835389

[ref6] Caravan P. Strategies for increasing the sensitivity of gadolinium based MRI contrast agents. Chem. Soc. Rev. 2006, 35, 512–523. 10.1039/b510982p. 16729145

[ref7] Caravan P. Protein-Targeted Gadolinium-Based Magnetic Resonance Imaging (MRI) Contrast Agents: Design and Mechanism of Action. Acc. Chem. Res. 2009, 42, 851–862. 10.1021/ar800220p. 19222207

[ref8] Abdukayum A.; Yang C.-X.; Zhao Q.; Chen J.-T.; Dong L.-X.; Yan X.-P. Gadolinium Complexes Functionalized Persistent Luminescent Nanoparticles as a Multimodal Probe for Near-Infrared Luminescence and Magnetic Resonance Imaging in Vivo. Anal. Chem. 2014, 86, 4096–4101. 10.1021/ac500644x. 24702120

[ref9] Riehemann K.; Schneider S. W.; Luger T. A.; Godin B.; Ferrari M.; Fuchs H. Nanomedicine--challenge and perspectives. Angew. Chem., Int. Ed. 2009, 48, 872–897. 10.1002/anie.200802585. PMC4175737 19142939

[ref10] Shi J.; Votruba A. R.; Farokhzad O. C.; Langer R. Nanotechnology in Drug Delivery and Tissue Engineering: From Discovery to Applications. Nano Lett. 2010, 10, 3223–3230. 10.1021/nl102184c. 20726522 PMC2935937

[ref11] Chen G.; Roy I.; Yang C.; Prasad P. N. Nanochemistry and Nanomedicine for Nanoparticle-based Diagnostics and Therapy. Chem. Rev. 2016, 116, 2826–2885. 10.1021/acs.chemrev.5b00148. 26799741

[ref12] Bagalkot V.; Zhang L.; Levy-Nissenbaum E.; Jon S.; Kantoff P. W.; Langer R.; Farokhzad O. C. Quantum Dot–Aptamer Conjugates for Synchronous Cancer Imaging, Therapy, and Sensing of Drug Delivery Based on Bi-Fluorescence Resonance Energy Transfer. Nano Lett. 2007, 7, 3065–3070. 10.1021/nl071546n. 17854227

[ref13] Lukianova-Hleb E. Y.; Hanna E. Y.; Hafner J. H.; Lapotko D. O. Tunable plasmonic nanobubbles for cell theranostics. Nanotechnology 2010, 21, 085102 10.1088/0957-4484/21/8/085102. PMC3074956 20097970

[ref14] Horcajada P.; Chalati T.; Serre C.; Gillet B.; Sebrie C.; Baati T.; Eubank J. F.; Heurtaux D.; Clayette P.; Kreuz C.; Chang J.-S.; Hwang Y. K.; Marsaud V.; Bories P.-N.; Cynober L.; Gil S.; Férey G.; Couvreur P.; Gref R. Porous metal–organic-framework nanoscale carriers as a potential platform for drug delivery and imaging. Nat. Mater. 2010, 9, 172–178. 10.1038/nmat2608. 20010827

[ref15] Eriksson P.; Tal A. A.; Skallberg A.; Brommesson C.; Hu Z.; Boyd R. D.; Olovsson W.; Fairley N.; Abrikosov I. A.; Zhang X.; Uvdal K. Cerium oxide nanoparticles with antioxidant capabilities and gadolinium integration for MRI contrast enhancement. Sci. Rep. 2018, 8, 6999 10.1038/s41598-018-25390-z. 29725117 PMC5934375

[ref16] Xu C.; Qu X. Cerium oxide nanoparticle: a remarkably versatile rare earth nanomaterial for biological applications. NPG Asia Mater. 2014, 6, e90 10.1038/am.2013.88.

[ref17] Celardo I.; Pedersen J. Z.; Traversa E.; Ghibelli L. Pharmacological potential of cerium oxide nanoparticles. Nanoscale 2011, 3, 1411–1420. 10.1039/c0nr00875c. 21369578

[ref18] Lee S. S.; Song W.; Cho M.; Puppala H. L.; Nguyen P.; Zhu H.; Segatori L.; Colvin V. L. Antioxidant Properties of Cerium Oxide Nanocrystals as a Function of Nanocrystal Diameter and Surface Coating. ACS Nano 2013, 7, 9693–9703. 10.1021/nn4026806. 24079896

[ref19] Asati A.; Santra S.; Kaittanis C.; Perez J. M. Surface-Charge-Dependent Cell Localization and Cytotoxicity of Cerium Oxide Nanoparticles. ACS Nano 2010, 4, 5321–5331. 10.1021/nn100816s. 20690607 PMC2947560

[ref20] Das S.; Dowding J. M.; Klump K. E.; McGinnis J. F.; Self W.; Seal S. Cerium oxide nanoparticles: applications and prospects in nanomedicine. Nanomedicine 2013, 8, 1483–1508. 10.2217/nnm.13.133. 23987111

[ref21] Celardo I.; De Nicola M.; Mandoli C.; Pedersen J. Z.; Traversa E.; Ghibelli L. Ce(3)^+^ ions determine redox-dependent anti-apoptotic effect of cerium oxide nanoparticles. ACS Nano 2011, 5, 4537–4549. 10.1021/nn200126a. 21612305

[ref22] Chen H.-I.; Chang H.-Y. Synthesis of nanocrystalline cerium oxide particles by the precipitation method. Ceram. Int. 2005, 31, 795–802. 10.1016/j.ceramint.2004.09.006.

[ref23] Patterson A. L. The Scherrer Formula for X-Ray Particle Size Determination. Phys. Rev. 1939, 56, 978–982. 10.1103/PhysRev.56.978.

[ref24] Deshpande S.; Patil S.; Kuchibhatla S. V. N. T.; Seal S. Size dependency variation in lattice parameter and valency states in nanocrystalline cerium oxide. Appl. Phys. Lett. 2005, 87, 133113 10.1063/1.2061873.

[ref25] Banerji A.; Grover V.; Sathe V.; Deb S. K.; Tyagi A. K. CeO2–Gd2O3 system: Unraveling of microscopic features by Raman spectroscopy. Solid State Commun. 2009, 149, 1689–1692. 10.1016/j.ssc.2009.06.045.

[ref26] Godinho M. J.; Gonçalves R. F.; S. Santos LP.; Varela J. A.; Longo E.; Leite E. R. Room temperature co-precipitation of nanocrystalline CeO2 and Ce0.8Gd0.2O1.9−δ powder. Mater. Lett. 2007, 61, 1904–1907. 10.1016/j.matlet.2006.07.152.

[ref27] Spanier J. E.; Robinson R. D.; Zhang F.; Chan S.-W.; Herman I. P. Size-dependent properties ofCeO2–ynanoparticles as studied by Raman scattering. Phys. Rev. B 2001, 64, 245407 10.1103/PhysRevB.64.245407.

[ref28] Garvie L. A. J.; Buseck P. R. Determination of Ce4+/Ce3+ in electron-beam-damaged CeO2 by electron energy-loss spectroscopy. J. Phys. Chem. Solids 1999, 60, 1943–1947. 10.1016/S0022-3697(99)00218-8.

[ref29] Caravan P.; Ellison J. J.; McMurry T. J.; Lauffer R. B. Gadolinium(III) Chelates as MRI Contrast Agents: Structure, Dynamics and Applications. Chem. Rev. 1999, 99, 2293–2352. 10.1021/cr980440x. 11749483

[ref30] Rohrer M.; Bauer H.; Mintorovitch J.; Requardt M.; Weinmann H. J. Comparison of magnetic properties of MRI contrast media solutions at different magnetic field strengths. Invest. Radiol. 2005, 40, 715–724. 10.1097/01.rli.0000184756.66360.d3. 16230904

[ref31] Wahsner J.; Gale E. M.; Rodríguez-Rodríguez A.; Caravan P. Chemistry of MRI Contrast Agents: Current Challenges and New Frontiers. Chem. Rev. 2019, 957–1057. 10.1021/acs.chemrev.8b00363. 30350585 PMC6516866

[ref32] Mullins D. R. The surface chemistry of cerium oxide. Surf. Sci. Rep. 2015, 70, 42–85. 10.1016/j.surfrep.2014.12.001.

[ref33] Wang Y.-J.; Dong H.; Lyu G.-M.; Zhang H.-Y.; Ke J.; Kang L.-Q.; Teng J.-L.; Sun L.-D.; Si R.; Zhang J.; Liu Y.-J.; Zhang Y.-W.; Huang Y.-H.; Yan C.-H. Engineering the defect state and reducibility of ceria based nanoparticles for improved anti-oxidation performance. Nanoscale 2015, 7, 13981–13990. 10.1039/C5NR02588E. 26228305

[ref34] Damatov D.; Mayer J. M. Hydro)peroxide ligands on colloidal cerium oxide nanoparticles. Chem. Commun. 2016, 52, 10281–10284. 10.1039/C6CC03790A. 27468991

[ref35] Baldim V.; Bedioui F.; Mignet N.; Margaill I.; Berret J. F. The enzyme-like catalytic activity of cerium oxide nanoparticles and its dependency on Ce3+ surface area concentration. Nanoscale 2018, 10, 6971–6980. 10.1039/C8NR00325D. 29610821

[ref36] Hu Z.; Ahrén M.; Selegård L.; Skoglund C.; Söderlind F.; Engström M.; Zhang X.; Uvdal K. Highly Water-Dispersible Surface-Modified Gd2O3 Nanoparticles for Potential Dual-Modal Bioimaging. Chem. - Eur. J. 2013, 19, 12658–12667. 10.1002/chem.201301687. 24175343

[ref37] Gupta A.; Das S.; Neal C. J.; Seal S. Controlling the surface chemistry of cerium oxide nanoparticles for biological applications. J. Mater. Chem. B 2016, 4, 3195–3202. 10.1039/C6TB00396F. 32263255

[ref38] Bêche E.; Charvin P.; Perarnau D.; Abanades S.; Flamant G. Ce 3d XPS investigation of cerium oxides and mixed cerium oxide (CexTiyOz). Surf. Interface Anal. 2008, 40, 264–267. 10.1002/sia.2686.

[ref39] Watanabe S.; Ma X.; Song C. Characterization of Structural and Surface Properties of Nanocrystalline TiO2–CeO2 Mixed Oxides by XRD, XPS, TPR, and TPD. J. Phys. Chem. C 2009, 113, 14249–14257. 10.1021/jp8110309.

[ref40] Baltrusaitis J.; Mendoza-Sanchez B.; Fernandez V.; Veenstra R.; Dukstiene N.; Roberts A.; Fairley N. Generalized molybdenum oxide surface chemical state XPS determination via informed amorphous sample model. Appl. Surf. Sci. 2015, 326, 151–161. 10.1016/j.apsusc.2014.11.077.

[ref41] Frick C.; Dietz A. C.; Merritt K.; Umbreit T. H.; Tomazic-Jezic V. J. Effects of prosthetic materials on the host immune response: evaluation of polymethyl-methacrylate (PMMA), polyethylene (PE), and polystyrene (PS) particles. J. Long-Term Eff. Med. Implants 2006, 16, 423–33. 10.1615/JLongTermEffMedImplants.v16.i6.20. 17956209

[ref42] Kim Y. K.; Chen E. Y.; Liu W. F. Biomolecular strategies to modulate the macrophage response to implanted materials. J. Mater. Chem. B 2016, 4, 1600–1609. 10.1039/C5TB01605C. 32263014

[ref43] Liu W. F.; Ma M.; Bratlie K. M.; Dang T. T.; Langer R.; Anderson D. G. Real-time in vivo detection of biomaterial-induced reactive oxygen species. Biomaterials 2011, 32, 1796–1801. 10.1016/j.biomaterials.2010.11.029. 21146868 PMC4130482

[ref44] Kaplan S. S.; Basford R. E.; Mora E.; Jeong M. H.; Simmons R. L. Biomaterial-induced alterations of neutrophil superoxide production. J. Biomed. Mater. Res. 1992, 26, 1039–1051. 10.1002/jbm.820260806. 1331115

