# Supplementary material for: Cerium Oxide Nanoparticles with Entrapped Gadolinium for High T1 Relaxivity and ROS-Scavenging Purposes
Source: ACS Omega. 2022 Jun 7;7(24):21337–45. doi: 10.1021/acsomega.2c03055 (PMC9218977; doi:10.1021/acsomega.2c03055)
Supplement: Supplementary file 1 — ao2c03055_si_001.pdf [file ao2c03055_si_001.pdf]

**Supporting Information**

# Cerium Oxide Nanoparticles with Entrapped Gadolinium for High $T_1$ Relaxivity and ROS-scavenging Purposes

*Peter Eriksson[1], Anh H.T. Truong[2], Caroline Brommesson[1], Anna du Rietz[1], Ganesh R. Kokil[3], Robert D. Boyd[4], Zhangjun Hu[1], Tram T. Dang[5] Per O.Å. Persson[5] and Kajsa Uvdal*[1]*

[1]Division of Molecular Surface Physics and Nanoscience, Department of Physics, Chemistry and Biology (IFM), Linköping University, SE-581 83 Linköping, Sweden

[2] Laboratory of Therapeutic Cellular and Drug Delivery Systems, School of Chemical and Biomedical Engineering (SCBE), Nanyang Technological University, 637459 Singapore

[3] School of Pharmacy, Pharmacy Australia Centre of Excellence, The University of Queensland, Brisbane, QLD Australia

[4] Division of Plasma Coatings Physics Department of Physics, Chemistry and Biology (IFM), Linköping University, SE-581 83 Linköping, Sweden

[5] Division of Thin Film Physics, Department of Physics, Chemistry and Biology (IFM), Linköping University, SE-581 83 Linköping, Sweden

* Email kajsa.uvdal@liu.se Tel: +46 734607571

**Contents of the material supplied as Supporting Information.**


- Zeta potential and Dynamic Light Scattering
- UV-VIS Absorbance
- Three-level phantom model for MRI studies

## Zeta potential and Dynamic Light Scattering

The Zeta potential was measured for the as-prepared Gd-CeNPs and the results are displayed in Figure S1. All Gd-CeNPs have Zeta potential in equal potential range above 30 mV, indicating good colloidal stability for all the prepared nanoparticles.

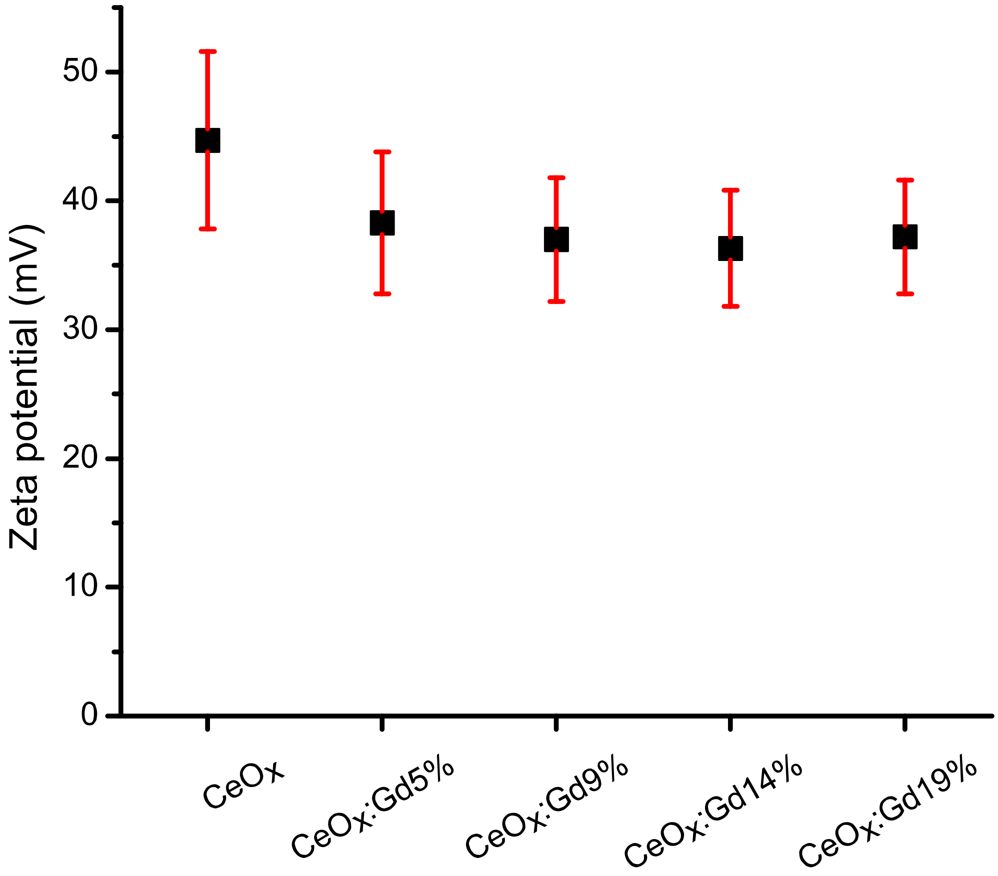


Figure S1. Measured Zeta potential of $CeO_x$:Gd0-19%

The number weighted hydrodynamic diameter was evaluated using dynamic light scattering (DLS), and the results are presented in Figure S2. DLS results indicate that all the Gd-CeNPs have a hydrodynamic diameter less than 7 nm. These diameters are reasonable comparing with the XRD data.

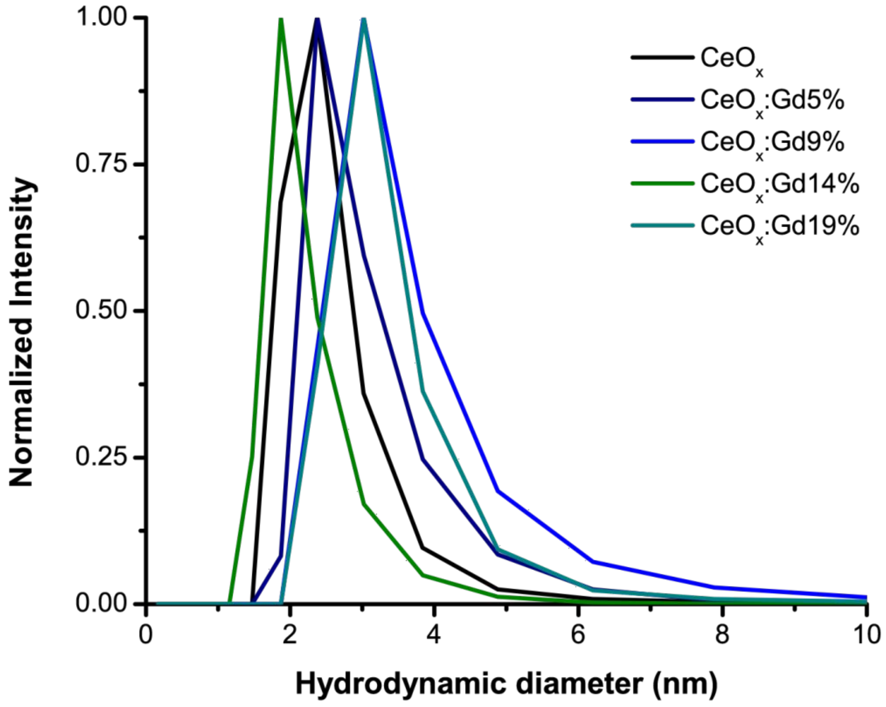


Figure S 2 Measured number weighted hydrodynamic diameters of $CeO_x$:Gd0-19%

**UV-VIS Absorbance**

The red-shift in absorbance for the different prepared Gd-CeNPs ([Ce] =50 µM) were estimated for different concentrations of $H_2O_2$ at optical density 0.05. An example is showed in Figure S3, where we have treated $CeO_x$ with 1 µM $H_2O_2$ to Δλ = 69.5 nm.

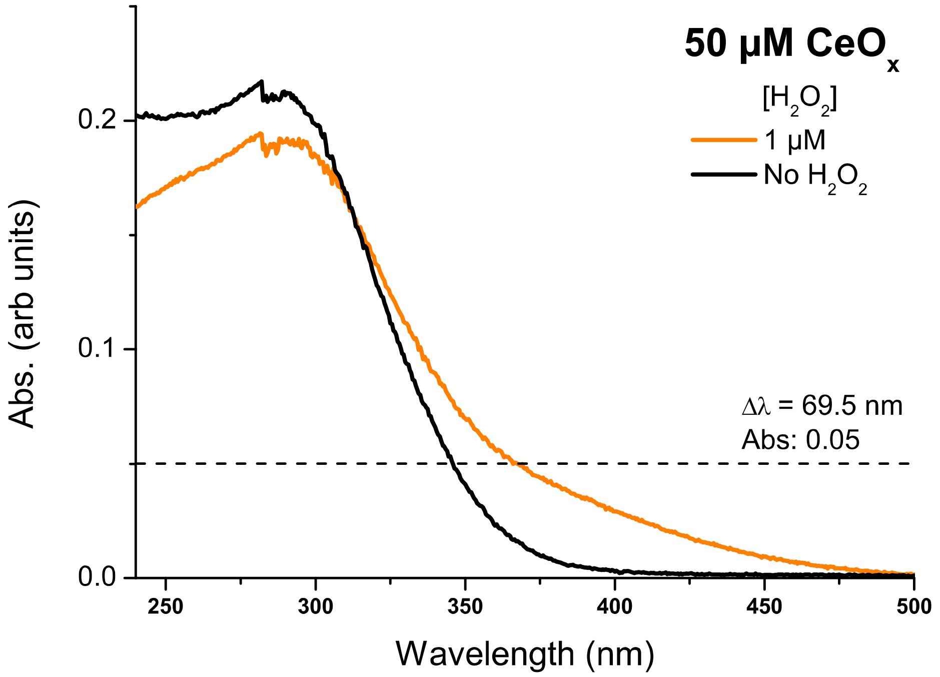


Figure S3 The red-shift (Δλ) was measured at the difference in wavelength at optical density 0.05 for sample, treated and untreated with $H_2O_2$. In this representative example, $CeO_x$ was treated with 1 µM $H_2O_2$ had a Δλ = 69.5 nm.

**Three-level phantom model for MRI studies**

The three-level phantom model that was used in magnetic resonance imaging (MRI) experiments is presented in Figure S4.

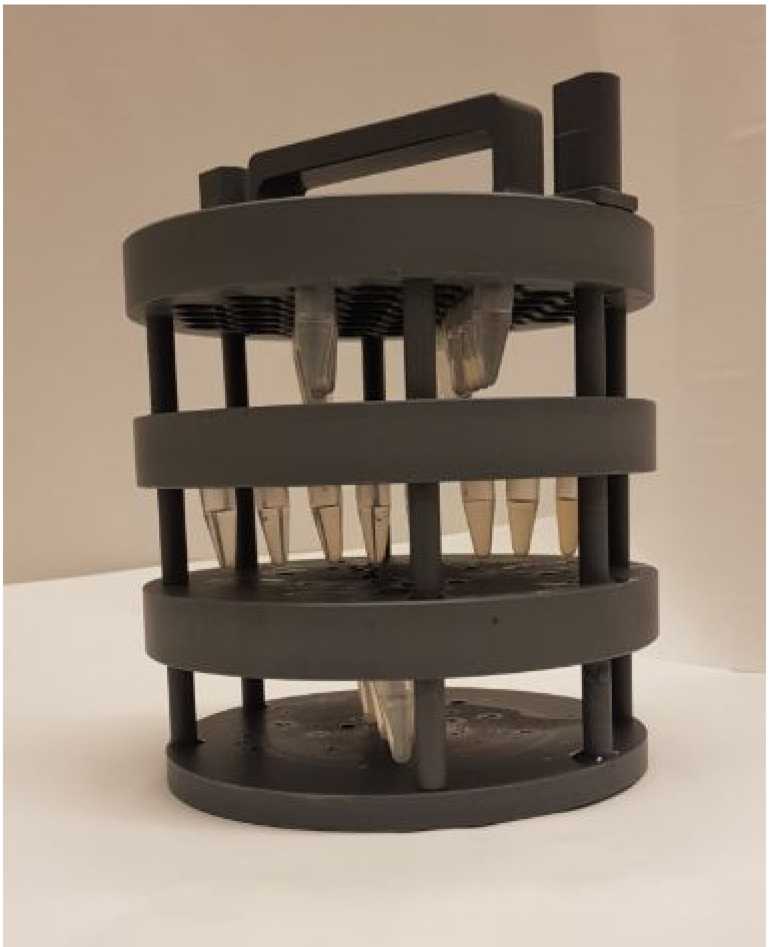

Figure S4. The three-level phantom model used in the MRI-experiments. Photograph courtesy of first author, P. Eriksson. Copyright 2020.
